# Retention Effects of Long-Term Balance Training with Vibrotactile Sensory Augmentation in Healthy Older Adults

**DOI:** 10.3390/s22083014

**Published:** 2022-04-14

**Authors:** Tian Bao, Fatemeh Noohi, Catherine Kinnaird, Wendy J. Carender, Vincent J. Barone, Geeta Peethambaran, Susan L. Whitney, Rachael D. Seidler, Kathleen H. Sienko

**Affiliations:** 1 Department of Mechanical Engineering, University of Michigan, Ann Arbor, MI 48109, USA; baotian@umich.edu (T.B.); catherinekinnaird@gmail.com (C.K.); vbarone@umich.edu (V.J.B.); 2 Department of Psychology, School of Kinesiology, University of Michigan, Ann Arbor, MI 48109, USA; fatemeh.noohibezanjani@ucsf.edu; 3 Department of Otolaryngology, Michigan Medicine, University of Michigan, 1500 E Medical Center Dr., Ann Arbor, MI 48109, USA; wcaren@med.umich.edu; 4 Physical Medicine & Rehabilitation, Michigan Medicine, University of Michigan, 1500 E Medical Center Dr., Ann Arbor, MI 48109, USA; geet@med.umich.edu; 5 Department of Physical Therapy and Otolaryngology, School of Health and Rehabilitation Sciences, University of Pittsburgh, 100 Bridgeside Point, Pittsburgh, PA 15219, USA; whitney@pitt.edu

**Keywords:** balance rehabilitation, vibrotactile, sensory augmentation, retention, older adults, home-based, telerehabilitation, telehealth, wearable device, sensory reweighting

## Abstract

Vibrotactile sensory augmentation (SA) decreases postural sway during real-time use; however, limited studies have investigated the long-term effects of training with SA. This study assessed the retention effects of long-term balance training with and without vibrotactile SA among community-dwelling healthy older adults, and explored brain-related changes due to training with SA. Sixteen participants were randomly assigned to the experimental group (EG) or control group (CG), and trained in their homes for eight weeks using smart-phone balance trainers. The EG received vibrotactile SA. Balance performance was assessed before, and one week, one month, and six months after training. Functional MRI (fMRI) was recorded before and one week after training for four participants who received vestibular stimulation. Both groups demonstrated significant improvement of SOT composite and MiniBESTest scores, and increased vestibular reliance. Only the EG maintained a minimal detectable change of 8 points in SOT scores six months post-training and greater improvements than the CG in MiniBESTest scores one month post-training. The fMRI results revealed a shift from activation in the vestibular cortex pre-training to increased activity in the brainstem and cerebellum post-training. These findings showed that additional balance improvements were maintained for up to six months post-training with vibrotactile SA for community-dwelling healthy older adults.

## 1. Introduction

Medical costs associated with age-related falls exceed $50 billion per year [1]. Exercise programs with targeted balance and strength training have been shown to improve balance and reduce falls among community-dwelling older adults [2,3,4]. Supervised training programs are typically individually tailored and lead to better outcomes as compared to class-based balance programs but are costly and not universally accessible [5]. While independent in-home programs may address these issues, the lack of clinician guidance results in fewer clinical improvements than supervised programs [6]. Telerehabilitation technologies may address the need for intensive, accessible, in-home semi-supervised balance training. Technologies, such as the Wii-fit [7], Kinect [8], and wearables that provide feedback [9] or incentives [10], have been investigated in combination with balance training. Sustained quality of life improvements and fewer fall incidents require retention of balance improvements.

Vibrotactile sensory augmentation (SA) systems have been used in balance-related research studies to estimate body motion and provide postural corrective cues in the form of vibration to the user. Multiple studies (although many were uncontrolled) have demonstrated short-term retentive (or possibly habituation or context-specific adaptation) effects of training with vibrotactile SA [11,12,13,14,15,16]. For example, Basta et al. showed that participants with balance deficits reduced their trunk sway after two weeks of training with vibrotactile SA and retained the effects of training for three months [12]. Kingma et al. reported improved mobility and balance scores in a small group of participants with bilateral vestibular loss who wore a vibrotactile belt daily for one month while standing and moving during activities of daily living [15].

A limited number of controlled studies have examined retention and/or carryover effects following longer-term training with SA. In a randomized controlled study, people with Parkinson’s disease participated in 12 sessions of clinical balance training [17]. The authors compared the effects of virtual reality (VR) augmented balance training using a dynamic balance board (VR group) to conventional balance training [17]. The VR group improved significantly on the Computerized Dynamic Posturography (CDP) Sensory Organization Test (SOT) condition 6 (unreliable vision and somatosensory inputs) as assessed within seven days after training; however, this finding was not significant at the four-week follow-up, suggesting limited retention effects. In another study involving people with Parkinson’s disease, improvements in SOT scores were retained and falls were reduced three months after 10 training sessions with vibrotactile SA over a two-week period [18]. Although this study did not have a control group, the SOT scores of the participants in this study showed greater improvement compared with 10 participants with Parkinson’s disease previously trained using CDP in a different study.

In a study involving older adults, balance training with vibrotactile SA three times per week for two weeks had minimal additional effects on both immediate and longer-term balance outcomes compared to a control group that performed balance training without vibrotactile SA [9]. However, in this study, balance tasks were not customized on an individual participant basis and the limited training period did not follow the recommended FITT principles of frequency, intensity, type, and time [19]. 

In a six-week study (totaling 18 balance training sessions) involving vestibular rehabilitation exercises, participants with vestibular deficits, regardless of group, demonstrated improvements in a subset of clinical and balance metrics immediately following completion of the balance training protocol [20]. However, the experimental group that trained with vibrotactile SA showed significantly greater improvements than the control group who trained without vibrotactile SA on the Activities-specific Balance Confidence Scale and postural stability during two standing balance exercises with head movements. 

Our prior work investigated the effects of long-term (eight-week home-based balance training program) balance training with and without vibrotactile SA on clinical outcome measures for community-dwelling older adults [21]. Participants who completed training with vibrotactile SA had greater improvements in SOT scores, Mini Balance Evaluation Systems Test scores, and Five Times Sit to Stand Test duration compared with the control group who trained without vibrotactile SA. Both groups also demonstrated increased vestibular reliance as measured by a ratio of SOT scores [21].

Beyond the lack of studies on retention effects, a limited number of studies have been performed to investigate the changes in the sensorimotor brain regions during and following balance training with SA. Multiple hypotheses have been posited to explain the potential mechanisms underlying balance improvements during and following training with SA devices, including sensory reweighting and context-specific adaptation [14,22]. Among the most relevant, a previous study reported that SA coupled with navigation training was associated with changes in brain activity in the sensorimotor and navigation (hippocampus, caudate) brain regions [23]. However, it is unknown whether brain-related changes occur after balance training with SA.

Given the lack of long-term training studies that explore the retention effects of training with SA and the lack of studies that have evaluated potential brain-related changes, there is a need for additional research that investigates the retention of training effects following longer-term training with vibrotactile SA. The two main purposes of this study were to (1) understand the retention effects of balance improvements by examining the balance performance following completion of an eight-week in-home balance training program with wearable vibrotactile sensory augmentation (SA) [21]; and (2) further the understanding of mechanisms underlying the balance improvements by examining brain changes in processing vestibular stimulation from pre- to post-training with SA. This study is one of the first of its kind to assess balance improvements and retention effects on a battery of clinical outcome measures after a long-term customized balance training program with and without SA.

## 2. Materials and Methods

### 2.1. Participants

**Figure 1:**
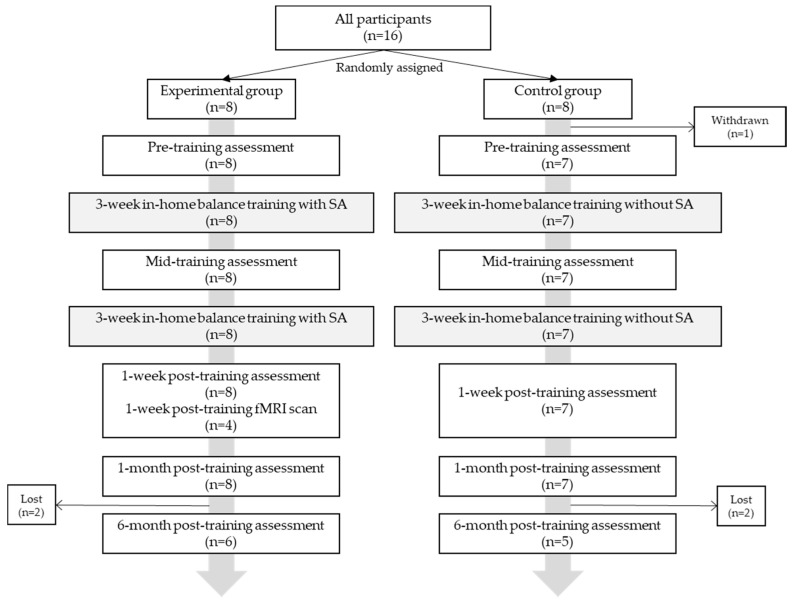
Participant allocation and experimental protocol.

In total, 16 healthy older adults (5 M, 75.4 ± 4.7 years) were recruited from the community. Participants were eligible if they were 65–85 years old, in good general health, and had self-reported balance concerns [21]. Participants were randomly allocated to either a control group (CG, *n* = 8) or experimental group (EG, *n* = 8). One participant was withdrawn due to an unrelated orthopedic condition, and four were lost to the six-month follow-up (Figure 1). All participants gave written informed consent, and the study protocol was approved by the University of Michigan Institutional Review Board (HUM00086479) and adhered to the Declaration of Helsinki.

### 2.2. Protocol

Participants performed eight weeks of in-home balance training (24 sessions, *n* = 15) and five clinical balance testing (CBT) sessions throughout the study: pre—(*n* = 15), mid—(*n* = 15), one week post—(*n* = 15), one month post—(*n* = 15), and six months post-training (*n* = 11) (Figure 1). CBT was performed by a licensed physical therapist blinded to the group and included: Computerized Dynamic Posturography (CDP) (Sensory Organization Tests (SOT)), Activity-specific Balance Confidence (ABC) Scale, Mini Balance Evaluations Systems Test (Mini-BESTest28 and Mini-BESTest32), Five Times Sit to Stand Test (5xSST), Four-Square Step Test (FSST), Functional Reach Test (FRT), 10-m walk test (self-selected and fast gait speeds), Timed Up and Go (TUG), and Timed up and Go–Cognitive (TUG-COG). Somatosensory, visual, and vestibular reliance were calculated as ratios of individual SOT scores [21,24]. The minimal detectable change (MDC) was determined for functional measures. MDC is an estimate of the smallest change in an outcome score that is correlated with a change in ability [25].

**Figure 2:**
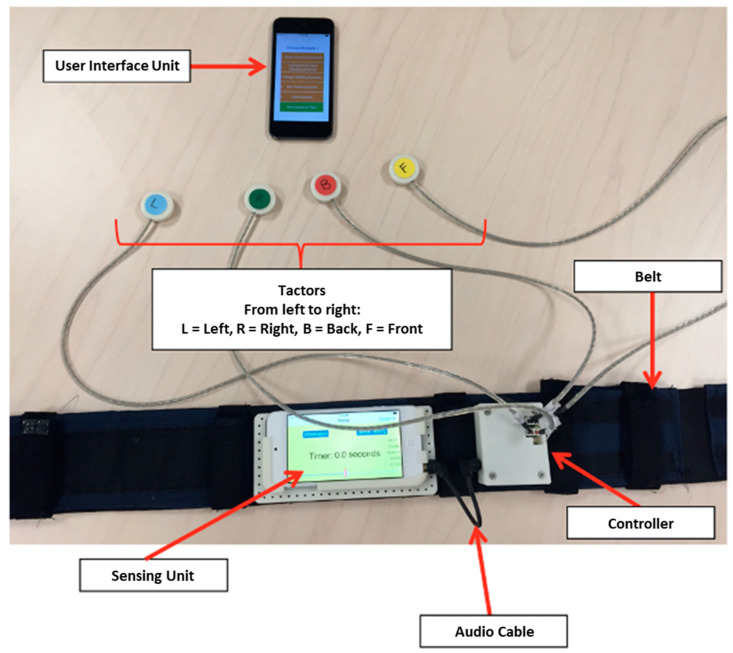
Smartphone-based balance trainer.

All participants wore a smartphone-based balance trainer comprising two Apple iPods (6th generation iPod touch, 2015), an elastic belt, and a customized tactor accessory during training, which has been described in detail in a prior publication (Figure 2) [21]. One of the two iPods that served as the sensing unit was attached to the elastic belt, which was worn around the torso at approximately the L4/L5 level to measure trunk sway; the second iPod served as the user interface unit attached to a lanyard and was worn around the neck. Participants in the EG received vibrotactile cues on their navel, spine, and left and right sides of their torso when their trunk motion (combination of angular position and angular velocity) exceeded preset thresholds. The preset thresholds for each exercise type (details below) were informed by the study team expertise and the thresholds used in previously published studies [20,21,26]. Participants were instructed to “move away from the vibration”. The gravitational outputs (*Class CoreMotion*, Apple Inc., Cupertino, CA, USA) from the torso-mounted iPod’s (i.e., sensing unit) accelerometers were used to estimate angular displacements (tilt angles) in the anterior-posterior and medial-lateral directions based on an algorithm developed by Lee et al. [27]. Angular velocities were measured from the sensing unit’s gyroscopes; both accelerometers and gyroscopes were sampled at 50 Hz. The customized tactor accessory included four tactors (Precision Microdrives™, 310–101 vibration motors encased in plastic housings [27]) that interfaced with a PCB-designed controller board and were powered by a 3.7 V battery. The controller board analyzed audio signals provided by the sensing unit and activated the corresponding tactor to provide vibrotactile cues.

Each hour-long balance training session consisted of six repetitions of six types of balance exercises from the following five exercise categories: static standing on firm and compliant surfaces, weight shifting, modified center of gravity (arm raises), and gait [21,28]. Other important exercise variables included eyes open/closed conditions and the addition of head movements. Vibrotactile cues were not provided for gait-based exercises. Participants were progressed through each category remotely by a physical therapist using the participants’ reported perceived stability scores [21], with the goal of providing a continuum of moderately challenging exercises using a progression protocol [28].

Functional magnetic resonance imaging (fMRI) was acquired from four EG participants using a 3.0 T MRI scanner (GE DISCOVERY MR750) one week post-training to investigate changes in brain function due to balance training. First, a whole brain structural image was acquired using a T1-weighted interleaved echo-planar imaging (EPI) sequence (TR = 12.2 s, TE = 5.1 ms, FA = 15°, matrix size = 256 × 256, FOV = 260 × 260 mm, slice thickness = 1 mm). Next, a gradient-echo spiral-pulse sequence (FOV = 220 mm, TR = 2 s, TE = 30 ms, number of slices = 43, voxel size = 3.4375 × 3.4375 mm) was used to acquire functional images. The participants’ head movements inside the scanner were minimized by a Velcro strap placed over their foreheads and padding placed around the sides of their heads. Participants’ physiological responses were collected using a pulse oximeter placed on their index fingers, and a respirometer wrapped around their abdomens. Low-force skull taps were applied over participants’ lateral cheekbones to stimulate the vestibular system using a pneumatic pulse system (Pneumatic Tactile Pulse System, Engineering Acoustics, Inc., Casselberry, FL, USA). Our prior work has shown that this system activates the vestibular cortical region and that resulting brain activity is correlated with balance under a variety of conditions [29,30]. A block design was implemented for the duration of each stimulation run (4 min) to include five alternating periods of rest (20 s) and stimulation (24 s) [29,30]. 

### 2.3. Analysis

All CBT outcome measures are shown as group mean values with standard errors of the means. Differences from pre- to post-training (one week post-, one month post-, six months post-training) were analyzed using a linear mixed model with time and the interaction between groups (EG vs. CG) as the main effects. Significance was set to 0.05.

The fMRI data preprocessing analyses were performed using spm8 software (The Wellcome Centre for Human Neuroimaging, London, UK) [31]. The raw data were examined for excessive motion as the skull vibration induced by the pneumatic taps could be a potential source of motion artifacts. Cut-off thresholds of >3 mm translation or >5° rotation of the head were implemented for head motion correction. The physiological responses (i.e., cardiac and respiration data) were regressed out of the functional data using the RETROICOR algorithm [32]. The first 10 volumes in each run were discarded to ensure the steady state of the MR signal at the beginning of the stimulation runs. Next, the functional images were realigned to the first functional volume of the run and the anatomical image. Both functional and anatomical images were then normalized to the Montreal Neurological Institute (MNI) template [33]. The cerebellum, however, was normalized to the Spatially Unbiased Atlas Template [32,34,35,36]. The normalized functional images were spatially smoothed with a Gaussian kernel function (8,8,8 mm). The smoothed functional images were then used to design the first-level analysis to compare brain activity during stimulation to rest. Next, a paired *t*-test was applied to compare brain activity pre- to post-training. A threshold of *p* ≤ 0.001 (unc.) and a minimum cluster size of 10 voxels (voxel size = 2 × 2 × 2 mm) were implemented for the results. The significant coordinates were localized using the MNI atlas [33] for the whole brain analyses, and the SUIT atlas [34] for the cerebellar coordinates. A gray matter inclusive mask was also applied using Automated Anatomical Labeling [37] to filter out activity in the white matter. To examine activity in regions previously identified as the vestibular nuclei (x = −16/16, y = −36, z = −32) [38], a small volume correction was applied, and the deep cerebellar nuclei were identified using the SUIT probabilistic atlas for deep cerebellar nuclei [35]. 

## 3. Results

### 3.1. Clinical Balance Testing Results

**Figure 3:**
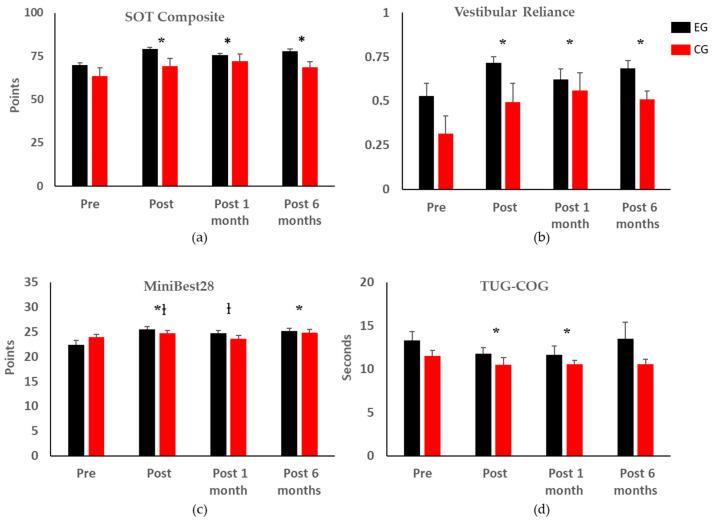
Statistical analysis of clinical balance test outcome metrics (* indicates significant differences with respect to the pre-training assessment regardless of group; † indicates significant differences between the two groups). Error bars represent the standard errors of the means. Only the clinical balance test outcome metrics with significant changes are shown; the complete results can be found in Appendix A. (**a**) SOT Composite: Sensory Organization Test Composite Score; (**b**) Vestibular Reliance calculated from the SOT; (**c**) Mini-BESTest28: Mini Balance Evaluations Systems Test with total score of 28; (**d**) TUG-COG: Timed up and Go with Cognitive Task.

SOT composite scores were significantly improved one week, one month, and six months post-training (*p* < 0.01, 0.01, and 0.01, respectively) regardless of group (Figure 3a). The EG demonstrated a mean minimal detectable change (MDC) of at least 8 points (healthy population [39]) for their SOT composite scores one week and six months post balance training (∆ = 8.1 ± 4.5, 9.2 ± 3.7 points, respectively) while the CG demonstrated a MDC one month post-training (∆ = 8.7 ± 4.2). There were no significant changes in the visual reliance scores, but vestibular reliance increased significantly one week, one month, and six months post-training (*p* < 0.001, 0.001, and 0.001, respectively), with no effect of group (Figure 3b). 

All participants had improved Mini-BESTest28 and Mini-BESTest32 scores one week and six months post-training (Mini-BESTest28: *p* < 0.001 and 0.01, respectively; Mini-BESTest32: *p* < 0.01 and 0.03, respectively); the EG had significantly better scores than the CG one week and one month post-training for the MiniBest28 (*p* = 0.04 and 0.03, respectively) and MiniBest32 (*p* = 0.01 and 0.05, respectively) (Figure 3c).

There were no changes in the TUG score, ABC score, self-selected, or fast gait speeds due to time or group. There was a significant improvement in TUG-COG one week and one month post-training (*p* = 0.04 and 0.03, respectively), with no difference between groups (Figure 3d). There was a significant improvement in the 5XSTS times one month post-training (*p* = 0.02), with no difference between groups. An MDC of 2.5 s (geriatric population) [40] was found in the EG one month post-training. There was a significant difference in the FSST six months post-training (*p* = 0.01), but no difference between groups. For the FRT, forward reach was reduced by 1.23 cm six months post-training (*p* = 0.01), with no difference between groups. Detailed data can be found in Appendix A.

### 3.2. fMRI Results

**Figure 4:**
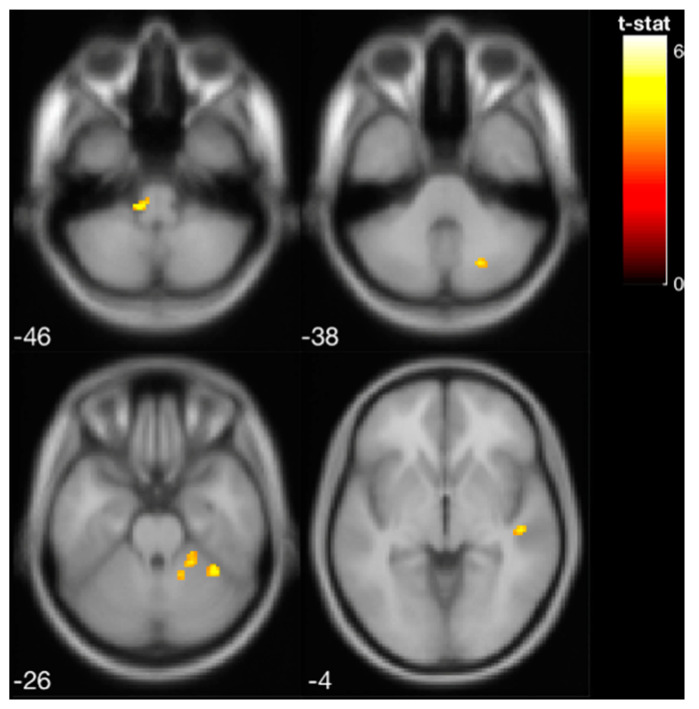
Increased brain activity in the brainstem and cerebellar cortex following balance training with vibrotactile SA (i.e., increased activation from pre- to post-training with vibrotactile SA) for the four EG participants that were scanned.

Pilot data from the four participants who underwent pre- and post-training fMRI of vestibular processing revealed a shift in activation from the vestibular cortex to the vestibular nucleus [41] in the brain stem and cerebellum immediately following the balance training with vibrotactile SA (Figure 4, Table 1).

## 4. Discussion

This study presents retention effects of an 8-week in-home balance training program with SA for healthy older adults. The results demonstrate improvements due to balance training regardless of group, with retention effects observed up to six months after completing the balancing training. However, only the EG demonstrated a minimal detectable change in SOT composite scores and 5XSTS scores. The EG also maintained a significantly higher improvement than the CG for the MiniBEST scores one month post-training. There were no significant changes in the TUG score, self-selected, or fast gait speeds, which may be due to ceiling effects or because SA was not provided during gait training tasks.

Balance training works by challenging the somatosensory, visual, and vestibular systems individually and by integrating multiple system inputs [42,43,44]. A subset of four EG participants underwent fMRI scans, and the results suggest functional reorganization of sensory processing and integration. We found that the pattern of brain activity in response to vestibular stimulation changed following training, exhibiting greater involvement of the brainstem and cerebellar regions. These findings suggest that SA modulates neural processing of vestibular stimulation, resulting in a shift from cortical to more sub-cortical regions, supporting the sensory reweighting mechanism theory [14]. This finding is comparable to that of Wildenberg et al., who reported an increase in activation of the brainstem and cerebellum after tongue-based electrotactile SA [45]. Moreover, a series of prior experiments by Wildenberg et al. investigated brain changes associated with long-term balance training with tongue electrotactile SA and found that balance-impaired individuals exhibited overactivation prior to training in comparison to controls in the occipital lobe and cerebellar vermis; these activity patterns were normalized following nine training sessions [46,47]. Evidence suggests that cerebellar processing of vestibular information contributes to self-motion perceptions [48,49,50]. It may be that SA coupled with balance training results in improved self-motion detection, linked to the increasing cerebellar activity we observed here. These results from a small subset of our participants should be followed up in future larger controlled fMRI studies.

It is compelling that both the brain and behavioral changes (an increase in vestibular reliance for both groups) suggest shifts in sensory reliance and integration with training [50]; participants increased their reliance upon vestibular inputs for balance following training. Older adults are generally more reliant on visual and somatosensory inputs and this work supports previous research indicating that progressive balance training may lead to sensory reweighting, resulting in increased vestibular reliance scores [51]. 

Given the small sample size, additional research is required to elucidate the improved functional balance outcomes with and without SA, and to correlate these with brain changes.

Balance training programs should incorporate different sensory conditions to promote the use of visual, vestibular, and proprioceptive inputs to target the individual’s deficits, but further research is needed on the optimal dosage and intensity by varying the training duration, training frequency, and SA activation thresholds. The data presented here indicate that while SA may provide some added benefit, the frequency and intensity via progressive customized balance training are important components of an in-home balance program, i.e., the FITT principle [52,53]. Retention effects were apparent six months post-training, but scores were lower than one week and one month post-training, indicating post intervention improvement wash-out. Progressively challenging balance training should be incorporated into an ongoing exercise program and lifestyle change to promote healthy aging and delay age-related balance declines.

## 5. Conclusions

Retention of balance training effects is imperative for fall prevention in the older adult population. Both groups (experimental group that received vibrotactile SA during balance training and the control group that completed balance training without vibrotactile SA) demonstrated significant improvements in their SOT composite and MiniBESTest scores, and increased vestibular reliance as determined by their SOT performance. However, only the group that trained with vibrotactile SA maintained a minimal detectable change of 8 points in their SOT scores six months following completion of the balance training protocol and greater improvements in their MiniBESTest scores one month following completion of the balance training protocol compared with the control group. Preliminary research suggests that SA may stimulate areas of the brain that facilitate the use of vestibular inputs for postural control. This sensory reweighting mechanism may be a critical component to consider when designing fall prevention programs.

## Figures and Tables

**Table 1:** Activated brain regions in response to vestibular stimulation at pre-training, post-training, and pre- vs. post-training (i.e., increased activation from pre- to post-training). Following balance training, the brain activity shifted from the vestibular cortex to the cerebellum and vestibular nucleus in the brainstem. The results are shown at *p* < 0.001 (unc.). MNI: Montreal Neurological Institute.

	Region Label	Extent	t-Value	MNI Coordinates		
				*x*	*y*	*z*
**Pre-training**	Parietal Operculum Cortex	72	9.983	−46	−36	22
**Post-training**	Cerebellar lobules I–IV	34	8.141	20	−44	−26
	Brainstem	102	7.528	0	−22	−16
	Middle Frontal Gyrus	14	6.453	48	6	46
	Middle Temporal Gyrus, temporooccipital part	22	6.331	−58	−48	−10
	Middle Frontal Gyrus	18	6.147	−34	6	32
	Inferior Frontal Gyrus, pars opercularis	10	5.918	−40	14	22
**Post-training > Pre-training**	Cerebellar lobules V–VI	40	6.408	36	−48	−28
	Cerebellar lobules I–IV	74	6.055	18	−44	−24
	Vestibular Nucleus in Brainstem	56	5.920	−12	−32	−42
	Superior Temporal Gyrus, posterior division	30	4.761	48	−22	−4
	Cerebellar lobule VIIB	28	4.695	22	−70	−38
	Cerebellar Crus I	22	4.289	−36	−54	−34

## Data Availability

The de-identified datasets generated and analyzed are available upon reasonable request.

## Appendix A

**Table A1:** Clinical balance testing results.

		Subject	1	2	3	4	5	6	7	8	9	10	11	12	13	14	16
	Time																
Gender			M	F	F	F	M	M	M	F	F	F	F	M	F	F	F
Age			83	83	70	72	70	80	73	70	82	78	74	74	74	73	75
Group			CG	EG	EG	CG	CG	EG	CG	EG	EG	CG	CG	EG	EG	EG	CG
SOT	Pre		71	63	78	49	76	68	60	83	74	83	68	58	50	74	43
	Post 1wk		81	83	76	46	73	86	65	85	79	85	76	60	79	65	67
	Post 1mo		83	76	78	49	77	78	65	85	77	85	77	65	73	62	75
	Post 6mo		80	77	-	56	75	76	69	85	76	-	71	61	76	-	-
MiniBESTest28	Pre		21	22	20	25	26	23	24	24	25	23	24	24	19	24	25
	Post 1wk		25	25	27	24	22	23	25	27	26	25	24	27	26	25	26
	Post 1mo		21	26	24	23	22	23	27	26	27	22	24	25	24	23	25
	Post 6mo		26	24	-	23	25	24	27	27	25	-	22	26	26	-	-
MiniBESTest32	Pre		24	26	23	28	30	25	26	27	28	26	27	28	21	28	29
	Post 1wk		27	29	31	27	24	26	28	31	29	27	26	31	30	29	30
	Post 1mo		25	29	27	26	25	25	31	30	30	26	26	29	27	28	29
	Post 6mo		30	26	-	27	29	26	30	30	28	-	24	30	30	-	-
ABC	Pre		95	92	94	95	95	94	98	86	93	89	92	94	87	98	96
	Post 1wk		92	82	89	-	93	87	99	98	97	91	94	83	95	98	93
	Post 1mo		89	88	88	-	92	81	99	99	96	91	96	93	96	98	96
	Post 6mo		93	91	-	88	93	86	99	99	98	-	91	88	94	-	-
TUG	Pre		9.9	8.4	9.3	9.3	9.3	13	8.6	9.8	13.6	10.5	12.4	10.6	10.7	10.1	10.5
	Post 1wk		9.8	9.9	10.4	8.8	9.5	12.8	9.7	8.9	11.4	12	9.3	10.3	9.6	9.7	13.2
	Post 1mo		9.3	10.3	9.1	8.2	9.9	12.2	8.4	7.2	13.5	10.5	10.1	13.4	8.5	8.4	13.7
	Post 6mo		9.8	9.3	-	10.1	10.2	15.5	8.7	8.1	9.6	-	11.2	10	9.1	-	-
TUG-COG	Pre		12.2	10	11.6	8.3	8.8	16.9	8.7	10.8	14.2	12.4	14.8	14.2	16.4	13.5	13
	Post 1wk		7.8	10.3	11.2	8.4	11.4	15	8.9	9.1	12.6	11.6	10.3	11.8	12.7	11.6	13.8
	Post 1mo		9.1	9.4	13.1	9.1	12	15.2	10.1	7.7	13.4	11	12.5	8.7	11.9	10.4	12.2
	Post 6mo		8.9	10.5	-	10.5	12	19.9	9.2	8.1	11	-	11.8	10.8	18.0	-	-
5TSTS	Pre		11	12	10.1	7.4	9.7	14.5	8.8	12.2	17.8	13.7	17.7	8.9	14.6	9.7	16.9
	Post 1wk		14.6	11	8.7	11	11.4	12.3	8.3	6.6	15	9.9	9.5	14.7	10.6	9.4	12.9
	Post 1mo		10.4	11.7	9.1	7.2	10	13.5	9.3	7.1	13.3	9.3	8.9	12.8	9.6	10.7	14.6
	Post 6mo		14.4	10.3	-	8.4	10.3	14.4	7.1	6.5	16.8	-	11.1	12.7	10.6	-	-
FSST	Pre		13	10.4	10.2	11	8.3	10.7	8.5	6.5	13	10.7	11.7	10.9	8.3	11.3	10.8
	Post 1wk		14.4	10.2	10.2	9.9	8.9	11	8.1	6.2	11.8	9.8	9.7	12.5	7.8	11.8	12.3
	Post 1mo		12.7	10.6	10.1	10.6	9.5	10.7	9	5.6	12.6	8.5	9.2	11.2	8.2	8.4	10.8
	Post 6mo		11.2	10.3	-	12.1	10.1	11.7	9.1	5.8	11.5	-	10.2	10	7.8	-	-
FRT	Pre		9.2	10.7	15.8	14.2	12.8	9.8	14.8	13.7	15.7	11.8	12.7	12	13.8	15.5	12
	Post 1wk		12	11.3	13.5	14.3	14.8	10.7	13.5	10.3	14.8	13.3	14.7	7.8	13.5	13.7	12
	Post 1mo		11.8	9.3	13.3	13.5	10.8	14.5	13.3	13	12.8	12.5	10	10.7	16	15.5	12.2
	Post 6mo		10.2	13.2	-	11.8	12	10.7	11.7	12.3	12.2	-	11.2	7.7	12.8	-	-
fMRI Scan					Yes		Yes	Yes		Yes					Yes		
